# The Overlooked Nucleocapsid Response: A Cohort Study of SARS-CoV-2 Vaccines in Brazil

**DOI:** 10.3390/pathogens14050445

**Published:** 2025-04-30

**Authors:** Fatima de Cássia Evangelista de Oliveira, Ana Carolina Matias Dinelly Pinto, Maria Francilene Souza Silva, Max Moreira Lizano Garcia, Maria da Conceição Rodrigues Fernandes, Gabriela Alexandria Damasceno, Amanda Campelo Lima de Melo, Tamires Cardoso Matsui, Tamiris de Fátima Goebel de Souza, Fernanda Gadelha Severino, Virgínia Angélica Silveira Reis, Caroline Passaes, Fernanda Montenegro de Carvalho Araújo, Luiz Odorico Monteiro de Andrade, Marcela Helena Gambim Fonseca

**Affiliations:** 1 Unidade de Apoio ao Diagnóstico da COVID-19, Fundação Oswaldo Cruz (Fiocruz), Eusébio 61773-270, Ceará, Brazil; carolina.dinelly@fiocruz.br (A.C.M.D.P.); lenolysilva@hotmail.com (M.F.S.S.); maxgarcia81@hotmail.com (M.M.L.G.); gabialexandria.ga@gmail.com (G.A.D.); amanda.campelo@gmail.com (A.C.L.d.M.); tamirescardoso@hotmail.com (T.C.M.); tamirisgsouza@gmail.com (T.d.F.G.d.S.); fernandamontenegrocaraujo@gmail.com (F.M.d.C.A.); odorico.monteiro@fiocruz.br (L.O.M.d.A.); marcela.gambim@fiocruz.br (M.H.G.F.); 2 Pasteur-Fiocruz Center on Immunology and Immunotherapy, Eusébio 61773-270, Ceará, Brazil; caroline.passaes@fiocruz.br; 3 Instituto de Saúde e Gestão Hospitalar (ISGH), Fortaleza 60843-070, Ceará, Brazil; fernandagadelha@isgh.org.br (F.G.S.); vlopessilveira@isgh.org.br (V.A.S.R.)

**Keywords:** SARS-CoV-2, CoronaVac, BNT162b2, healthcare workers, antibody response

## Abstract

SARS-CoV-2 has caused global disruptions, prompting studies on immune responses to COVID-19 vaccines, particularly antibodies against the Spike (S) protein. However, responses to the Nucleocapsid (N) protein remain less explored. This study evaluated whether CoronaVac induces anti-N antibodies, and analyzed antibody dynamics after a BNT162b2 booster, given that CoronaVac targets both S and N proteins, while BNT162b2 targets only the S protein. Serum samples were collected at multiple intervals post-vaccination. The percentage of participants with positive anti-N antibodies increased from 40.26% to 62.09% after two doses of CoronaVac, but declined over time, reaching 29.07% and 18.87% after the second and third doses, respectively. However, seropositivity rose to 43.48% three months after the booster. Anti-S antibody levels peaked at 31,394 AU/mL after the booster, compared to 723.4 AU/mL after the first dose. These findings indicate that CoronaVac stimulates antibody responses against both S and N proteins. Monitoring antibody dynamics is crucial for optimizing vaccination strategies, particularly for high-risk populations, to help control COVID-19.

## 1. Introduction

Severe acute respiratory syndrome coronavirus 2 (SARS-CoV-2), the virus behind coronavirus disease 2019 (COVID-19), first emerged in China in December 2019 and quickly spread worldwide, triggering a global pandemic with profound effects on public health and the economy [1]. The development of immunity against SARS-CoV-2 through vaccination was crucial in controlling the COVID-19 pandemic and mitigating its economic and public health impacts. A variety of conventional and innovative platforms were employed in developing COVID-19 vaccine candidates, including inactivated virus vaccines, non-replicating viral vector vaccines, RNA-based vaccines, DNA-based vaccines, and protein subunit recombinant vaccines. These diverse approaches elicited distinct immune responses by targeting different viral proteins, enhancing overall protection against SARS-CoV-2.

The structure of SARS-CoV-2 consists of a Nucleocapsid, which protects the viral genome, and an outer Envelope. The Nucleocapsid houses the viral genome, bound to the Nucleocapsid (N) protein, while the Envelope is composed of a phospholipid bilayer containing the following key structural proteins: Spike (S), Membrane (M), and Envelope (E) [2]. The S protein enables viral entry by binding to the host cell receptor, the M protein maintains Membrane curvature and interacts with the Nucleocapsid, the E protein is essential for viral assembly and release, and the N protein stabilizes the viral RNA genome [3,4]. In Brazil, healthcare workers (HCWs) were the first group to be vaccinated, receiving mainly CoronaVac for the primary vaccination and BNT162b2 as the booster dose. CoronaVac uses an inactivated whole SARS-CoV-2 virus, allowing for the induction of antibodies against both the Spike and Nucleocapsid proteins. In contrast, the Pfizer mRNA vaccine contains messenger RNA encoding the S1 subunit of the Spike protein, inducing the production of antibodies exclusively against this protein [5]. Following the implementation of the COVID-19 vaccination, most studies have assessed vaccine responses [6] primarily by measuring the magnitude of the Spike (S)-specific antibody response [7,8,9]. In contrast, significantly less attention has been given to immune responses targeting other SARS-CoV-2 proteins, such as the Nucleocapsid (N) protein, even in vaccination regimens that include live-attenuated virus vaccines. Our group has been monitoring antibody response profiles across different vaccination protocols. Previously, we reported the dynamics of anti-Spike (S) antibodies following CoronaVac immunization [10]. In this study, we assess whether CoronaVac induces the production of anti-Nucleocapsid (N) antibodies and examine the dynamics of both anti-S and anti-N antibodies after the BNT162b2 booster, particularly during the Omicron wave in Brazil.

## 2. Materials and Methods

### 2.1. Participants and Study Design

**Figure 1:**
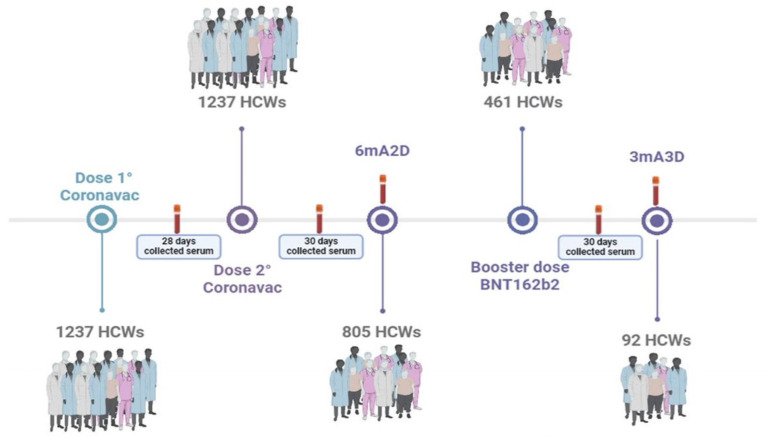
Schematic illustration of participant enrollment and sample collection. Serum samples were collected 28 days after the first dose (1D) of CoronaVac, 30 days (2D) and 6 months after the second dose of CoronaVac (6mA2D), and 30 days (3D) and 3 months (3mA3D) after the booster dose with the mRNA BNT162b2 vaccine.

This study included 1252 healthcare workers (HCWs) of both genders, aged 18 and above, who received two doses of the CoronaVac vaccine followed by a booster dose of the BNT162b2 mRNA vaccine (Pfizer-BioNTech). All participants provided informed consent under a protocol approved by the Ethics Committee of Hospital Geral Dr. César Cals (CAAE 39691420.7.0000.5049). HCWs were recruited from 29 healthcare institutions in Ceará, Brazil, including 25 public Brazilian Health System facilities and 4 private organizations. Individuals who were unvaccinated or unable to provide blood samples within the study’s designated timeframe were excluded. This study builds upon a previously published study, in which serum samples were collected 28 days after the first dose of CoronaVac (1D), 30 days after the second dose (2D), and six months post-second dose (6mA2D) [5]. Here, we included the following two additional serum collections: 30 days (3D) and three months (3mA3D) after the BNT162b2 booster (Figure 1). All participants completed a questionnaire, providing demographic and clinical information, and signed an informed consent form. Their history of COVID-19 infection was documented from before vaccination until the final sample collection. Blood samples were collected at participants’ workplaces and sent to the Serology Laboratory of the COVID-19 Diagnosis Support Unit at Fundação Oswaldo Cruz (Fiocruz Ceará), Brazil, for serological analysis.

### 2.2. Methods

All samples were tested for immunoglobulin G (IgG) against the Spike (S) protein and Nucleocapsid (N) protein of SARS-CoV-2, using chemiluminescent microparticle immunoassays (CMIA) from Abbott Diagnostics (Abbott Core Laboratory, SARS-CoV-2 Immunoassays: advancing diagnostics of COVID-19. Available at: https://www.corelaboratory.abbott/int/en/offerings/segments/infectious-disease/sars-cov-2.html, accessed on 1 January 2025), detected by the ARCHITECT i2000SR equipment (Abbott). For N IgG, the qualitative SARS-CoV-2 IgG I Assay (Ref 6R86) was used, which has a sensitivity of 100% and a specificity of 99.63%. For S IgG, the quantitative SARS-CoV-2 IgG II Quant Assay (Ref 6S60) was employed to measure antibodies targeting the receptor-binding domain (RBD) of the S1 subunit of the SARS-CoV-2 S protein. This assay shows 100% positive agreement with the plaque reduction neutralization test and has a sensitivity of 99.4% and a specificity of 99.6% [11]. As it is a qualitative test, the interpretation of the N IgG result was determined by an index (S/C) value, which represents the ratio over a threshold value. An index <1.4 was considered negative, and ≥1.4 was considered positive. For S IgG, antibody concentrations were expressed in arbitrary concentration units (AU/mL), where a value of ≥50 AU/mL was considered positive and <50 AU/mL was reported as negative. All stages of the test, as well as the construction of the graphs, were performed following the manufacturer’s instructions, adopting the cutoff point recommended by the manufacturer, being 1.4 for IgG against protein N and 50 for IgG against protein S (Abbott Core Laboratory).

### 2.3. Statistical Analyses

The data variables were considered non-normally distributed, and a statistical analysis was conducted, using medians and interquartile ranges (IQR) for continuous variables, and median, frequency, and percentage values for qualitative variables. Fisher’s exact test was used for analyzing qualitative variables. For S IgG levels, the Mann–Whitney test was employed to compare two groups within the same evaluation time, while the Kruskal–Wallis test with a subsequent Dunn’s multiple comparison test was used for group comparisons across different evaluation times. Analyses were performed, and scatter plots were generated using GraphPad Prism (version 8.01). A *p*-value of <0.05 was considered statistically significant.

## 3. Results

### 3.1. Longitudnal Analysis of Anti-N Antibody Responses Following Vaccination with CoronaVac/BNT162b2 Booster in a Cohort of Healthcare Workers (HCWs)

We initially aimed to evaluate anti-N IgG antibody responses in a cohort of 1237 healthcare workers (HCWs) at the following three key time points: 28 days after the first dose of the CoronaVac vaccine (1D), 30 days after the second dose (2D), and six months following the second dose (6mA2D), in 805 HCWs who remained in the study. Following the booster dose with the BNT162b2 vaccine, we were able to evaluate the anti-N IgG antibodies in 461 HCWs 30 days after the booster (3D). Three months after the booster dose (3mA3D), we assessed N IgG antibodies in 92 HCWs who continued to be in the study. The participants were between 20 and 73 years old, with a predominance of the 30 to 50 age group in all phases analyzed.

**Figure 2:**
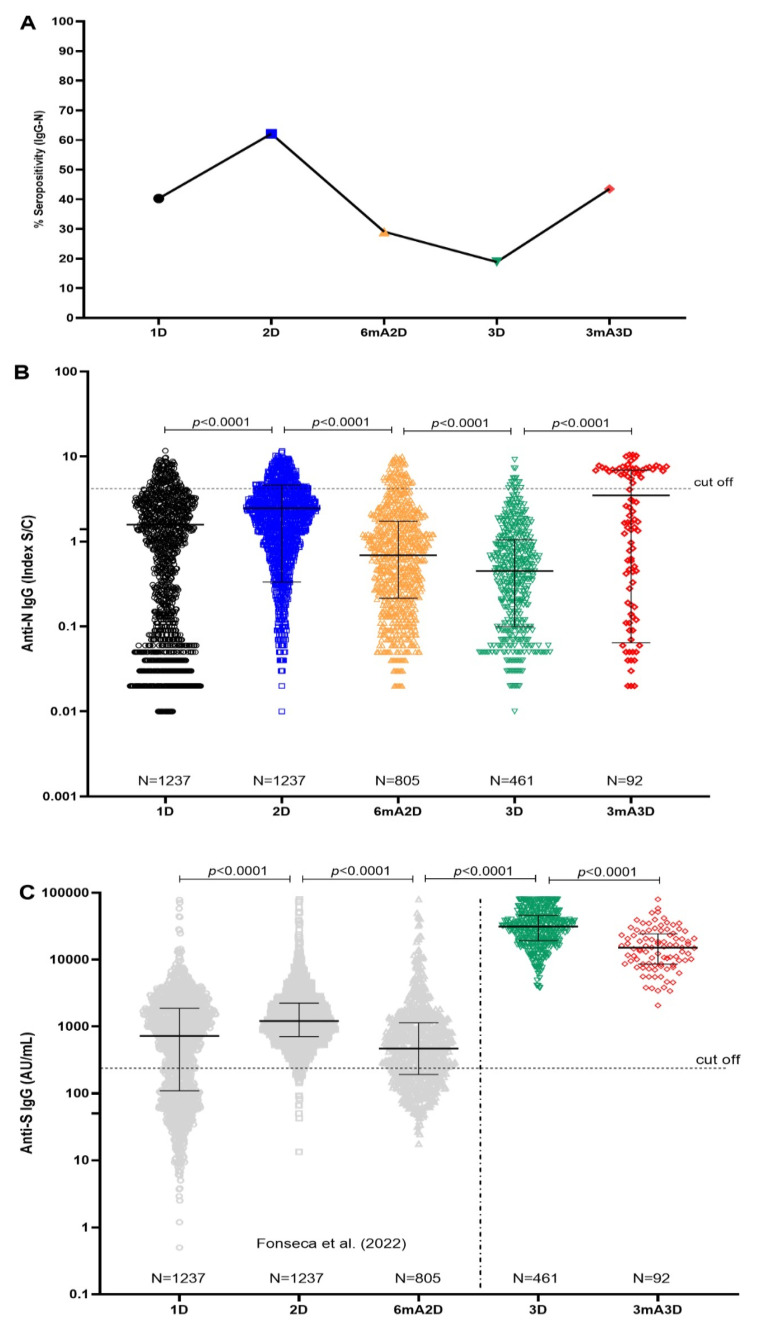
Antibody response over time in a cohort of healthcare workers vaccinated with two doses of the CoronaVac vaccine (https://www.sinovac.com), followed by a booster dose with the BNT162b2 vaccine (Pfizer-BioNTech, https://www.pfizer.com). (**A**) N IgG seropositivity. (**B**) N IgG levels. (**C**) S IgG levels. Data phases 1D, 2 D, and 6mA2D (1C) published by Fonseca et al. (2022) [10]. Antibody responses were evaluated 28 days after the first dose of the CoronaVac vaccine (1D); 30 days (2D) and 6 months (6mA2D) after the second dose of the CoronaVac vaccine; 30 days (3D) and 3 months (3mA3D) after the booster dose with the BNT162b2 vaccine. For panels (**B**,**C**), black lines indicate median levels’ values and error bars’ interquartile ranges; horizontal dotted lines indicate cutoff values. N, Nucleocapsid protein; S, Spike protein. The comparisons between groups of the same time point were performed using a *t*-test and Mann–Whitney. *p*-values < 0.0001 are reported as exact numbers. Data were expressed by AU/mL and S/C concentration, signal-to-cutoff ratio.

We observed that anti-N IgG antibodies were detectable in 40.26% of healthcare workers (HCWs) 28 days after the first dose of the CoronaVac vaccine (1D), and in 62.09% of HCWs 30 days after the second dose (2D). A continuous decline in seropositivity was noted at 6 months after the second dose (6mA2D), with a rate of 29.07%, and it further decreased to 18.87% at 30 days after the BNT162b2 booster dose (3D). However, seropositivity was partially restored to 43.48% at 3 months after the booster dose (3mA3D) (Figure 2A).

N IgG levels were significantly higher at 30 days after the second dose (2D) (median: 1.97; IQR: 0.85–3.425) compared to 28 days after the first dose (1D) (median: 0.87; IQR: 0.07–2.485, *p* < 0.0001). A significant and continuous decrease in N IgG levels was observed at 6 months post-second dose (6mA2D) (median: 0.69; IQR: 0.215–1.735, *p* < 0.0001). As expected, the booster vaccination with the BNT162b2 mRNA vaccine did not lead to a significant increase in anti-N IgG levels 30 days after vaccination (3D) (median: 0.45; IQR: 0.10–1.05, *p* < 0.0001). However, a substantial increase in N antibody levels was observed at 3 months post-booster (3mA3D) (median: 1.765; IQR: 0.1825–6.945), compared to the values measured at 30 days post-booster (3D) (*p* < 0.0001) (Figure 2B and Table 1).

### 3.2. Anti-S Antibody Responses Following mRNA BNT162b2 Booster in a Cohort of Healthcare Workers (HCWs)

Regarding the dynamics of anti-S IgG antibodies, we previously reported that S antibody levels increased after the second dose of the CoronaVac vaccine (2D), but waned six months after the second dose (6mA2D) (Fonseca et al., 2022) [10]. In the present study, we assessed anti-S IgG antibody levels 30 days after the booster dose (3D) in a cohort of 461 healthcare workers (HCWs), and three months later (3mA3D) in 92 HCWs who remained in the study (Figure 2A). Some factors, such as disinterest in continuing in the study, changing jobs, or not complying with the established vaccination schedule, may have contributed to the gradual reduction in participants. Although seropositivity was 100% at both time points, we observed a significant decrease in anti-S IgG titters at 3mA3D compared to 3D (*p* < 0.0001), with S IgG levels of 31,394 AU/mL and 15,107 AU/mL, respectively (Figure 2C and Table 1). It is of note that, at these timepoints, the cohort had a higher representation of female participants, with 363 (78.74%) females and 98 (21.26%) males at 3D, and 70 (76.08%) females and 22 (23.92%) males at 3mA3D. It is noteworthy that the cohort at both post-booster timepoints had a higher proportion of female participants, which may influence S-IgG levels. The antibody data corresponding to timepoints 1D, 2D, and 6mA2D for both S and N proteins were previously published [10] and are represented in the current study’s figures for context.

## 4. Discussion

In the present study, we assessed antibody responses against both the N and S SARS-CoV-2 proteins in a cohort of healthcare workers who were initially vaccinated with CoronaVac, an inactivated virus vaccine, and subsequently received a booster dose of the mRNA BNT162b2 vaccine (Pfizer/BioNTech), which targets the spike protein of the virus.

Antibodies specific to the viral S protein showed a significant increase after the booster dose with BNT162b2 (Pfizer/BioNTech), but a decline in antibody levels was observed three months later. Despite this decrease, seropositivity remained at 100%. The relationship between antibodies and immunity to SARS-CoV-2 infection is not fully understood and continues to be a subject of ongoing research. Naaber et al. (2021) [12] explained that antibody waning is expected, as not all vaccine-induced plasmablasts become or are sustained as long-lived memory B lymphocytes. They also emphasized that antibody titers do not necessarily correlate with protective immunity. In their study, neutralizing activity was maintained in healthcare workers (HCWs), even those with low IgG levels, suggesting that the threshold of antibody titers required for protection against reinfection is still unclear. Moreover, it is expected that, even with low antibody levels, memory B cells persist, enabling a faster humoral response during reinfections, which may result in a lower viral load and reduced damage to the host. Additionally, immune responses to SARS-CoV-2 are multifaceted, involving not only antibodies, but also other cell-mediated immune mechanisms.

CoronaVac stimulated the production of antibodies against both the viral Spike protein (as previously reported by our group [13]) and the Nucleocapsid protein [10]. As CoronaVac contains the entire inactivated virus, the immune system produces antibodies that target a wide array of SARS-CoV-2 proteins (antigens). In contrast, the BNT162b2 vaccine (Pfizer/BioNTech) was designed to prime the immune system to specifically recognize the S protein [14]. As a result, an increase in N antibodies was not observed after the BNT162b2 booster dose. This distinction highlights that CoronaVac promotes the generation of a broader antibody repertoire, which could be particularly relevant in the context of emerging coronavirus variants. This finding aligns with the research by Rosa and colleagues (2022) [15], who compared the immune responses elicited by the two major COVID-19 vaccines, BNT162b2 (mRNA) and CoronaVac (inactivated), in adolescents. Their study showed that BNT162b2 generated higher antibody levels than CoronaVac. However, both vaccines elicited robust T-cell responses specific to the S antigen, with only CoronaVac triggering T-cell responses to the N and M antigens. These results suggest potential differences in the durability and cross-protection of these vaccines against variants of concern.

Interestingly, a significant increase in N antibody levels was observed three months after the booster dose. This finding may be attributed to the Omicron wave in Brazil. The peak of the Omicron outbreak in Brazil occurred between November 2021 and March 2022 [16], while the cohort received the BNT162b2 booster in October 2021, one month before the Omicron surge. Notably, 10.86% (10/92) of healthcare workers (HCWs) reported a PCR-positive test for SARS-CoV-2 at the 3mA3D time point. Given that the Omicron variant carries mutations in the Spike protein that enable immune escape, vaccinated individuals may still experience an initial infection [17]. The high replication capacity of the Omicron variant could lead to the exposure of the internal N protein, which may trigger an N protein-specific antibody response. Furthermore, as the cohort had received the CoronaVac primary vaccination, whether CoronaVac generates long-term immunological memory specifically targeting the N protein—thereby contributing to an increase in N-IgG antibodies during SARS-CoV-2 reinfection—remains unclear.

In summary, this study demonstrated that CoronaVac induced S- and N-specific antibody responses. Furthermore, the S-IgG antibodies waned over time. Otherwise, the N-IgG antibodies increased after the Omicron wave in Brazil, reinforcing the role of N-specific antibodies as biomarkers of recent infection. Further studies are needed to clarify whether CoronaVac induces memory cells to the N protein and confers an advantage in clinical protection compared to other vaccines.

## 5. Conclusions

This study highlights that CoronaVac induces both S- and N-specific antibody responses, while the BNT162b2 booster significantly increases S-IgG levels without affecting N-IgG. The later rise in N-IgG, observed three months post-booster, likely reflects natural infection during the Omicron wave, highlighting N-IgG as a potential marker of recent SARS-CoV-2 exposure. These findings underscore the importance of monitoring both S and N antibody dynamics to better evaluate vaccine performance and population-level exposure, particularly in the context of newly emerging SARS-CoV-2 variants.

## Figures and Tables

**Table 1:** The median (and IQR) values of the antibody levels after the BNT162b2 booster shot in previous recipients of CoronaVac.

Phases	S IgGs	*p*-Value	N IgG	*p*-Value
1D	723.4 (109.6–1 875)		0.87 (0.07–2.485)	
2D	1208 (706.1–2 236)		1.97 (0.85–3.425)	<0.0001
6mA2D	453.1 (190.9–1 166)		0.69 (0.215–1.735)	<0.0001
3D	31394 (19 255–46 155)	-	0.45 (0.100–1.050)	<0.0001
3mA3D	15 107 (8 580–24 427)	<0.0001	1.765 (0.1825–6.945)	<0.0001

Abbreviations: 1D: 28 days after the first dose of CoronaVac vaccine; 30 days (2D) and 6 months (6mA2D) after the second dose of CoronaVac vaccine; 30 days (3D) and 3 months (3mA3D) after the booster dose with the BNT162b2 vaccine. N, Nucleocapsid protein; S, Spike protein. The comparisons between groups at the same timepoint were performed using a *t*-test and Mann–Whitney, significantly different (*p* < 0.05). S IgG levels. Data phases 1D, 2 D, and 6mA2D (1C) published by Fonseca et al. (2022) [10].

## Data Availability

The data that support the findings of this study are available from the corresponding author upon reasonable request.

## Abbreviations

The following abbreviations are used in this manuscript:

S	Spike
M	Membrane
E	Envelope
N	Nucleocapsid
HCWs	Health professionals
1D	28 days after the first dose
2D	30 days after the second dose
6mA2D	6 months after the second dose
3D	30 days after the booster dose
3mA3D	3 months after the booster dose
IgG	Immunoglobulin G
RBD	Receptor-binding domain
IQR	Interquartile range
